# Author Correction: Iron acquisition system of *Sphingobium* sp. strain SYK-6, a degrader of lignin-derived aromatic compounds

**DOI:** 10.1038/s41598-020-73843-1

**Published:** 2020-10-02

**Authors:** Masaya Fujita, Taichi Sakumoto, Kenta Tanatani, HongYang Yu, Kosuke Mori, Naofumi Kamimura, Eiji Masai

**Affiliations:** Department of Bioengineering, Nagaoka University of Technology, Nagaoka, Niigata 940-2188 Japan

Correction to: *Scientific Reports* 10.1038/s41598-020-68984-2, published online 22 July 2020

This Article contains an error in Table 1 in the Locus Tag column where,

“SLG_14330 (*tonB1*)”.

should read:

“SLG_14430 (*tonB1*)”.

