# Supplementary material for: Iron acquisition system of Sphingobium sp. strain SYK-6, a degrader of lignin-derived aromatic compounds
Source: Sci Rep. 2020 Jul 22;10:12177. doi: 10.1038/s41598-020-68984-2 (PMC7376174; doi:10.1038/s41598-020-68984-2)
Supplement: Supplementary file 1 — Supplementary Information. [file 41598_2020_68984_MOESM1_ESM.pdf]

Supplementary information

Iron acquisition system of *Sphingobium* sp. strain SYK-6, a degrader of lignin-derived aromatic compounds

Masaya Fujita, Taichi Sakumoto, Kenta Tanatani, HongYang Yu, Kosuke Mori, Naofumi Kamimura, and Eiji Masai*

Department of Bioengineering, Nagaoka University of Technology, Nagaoka, Niigata, Japan.

*Corresponding author:

Eiji Masai

Department of Bioengineering, Nagaoka University of Technology, Nagaoka, Niigata 940-2188, Japan

E-mail: emasai@vos.nagaokaut.ac.jp

Contents list

Supplementary tables: Table S1-S5
Supplementary figures: Fig. S1-S21
References for Supplementary information

Table S1. Fur box-like sequences upstream of *tonB2*, TBDR genes, and *feo* in SYK-6

Locus tag	Putative function	Accession number	Fur box-like sequence	Position (from start codon)
SLG_04320–04360	TBDR	BAK65109.1	GCTGCAACTCACTATCAAT	−37 to −19
SLG_04380	TBDR	BAK65113.1	AATGCGAATCATTCGCATT	−121 to −103
SLG_10860	TBDR	BAK65761.1	AATGATATTAACTCGCAAT	−61 to −43
SLG_17010	TBDR	BAK66376.1	GTTGCGACTAACTCGCATT	−43 to −25
SLG_34540 (*tonB2*)	TonB	BAK68129.1	TATGCGAATGACTCGCAAT	−47 to −29
SLG_34550 (*fiuA*)	TBDR	BAL68130.1	GTTGCGAATGATTCTCACT	−89 to −71
SLG_36850–36840 (*feoAB*)	Feo	BAK68359.1	ATTGATAATCACTCGCATC	−39 to −21

Table S2. SYK-6 proteins showing homology with known iron transporters

Protein	Function[a]	Substrate	Accession no.	Species and strain	Similar proteins in SYK-6	Sequence identity (%)[b]	Putative function	Reference
**Inner membrane**								
FepB	SBP	Enterobactin	P0AEL6	*Escherichia coli* K-12	–	–	–	1
FhuD	SBP	Ferrichrome	P07822	*Escherichia coli* K-12	–	–	–	2
FecB	SBP	Ferric citrate	P15028	*Escherichia coli* K-12	–	–	–	3
SirA	SBP	Staphyloferrin B	BAB41330	*Staphylococcus aureus* N315	–	–	–	4
FatB	SBP	Ferric anguibactin	P11460	*Vibrio anguillarum* 775	–	–	–	5
PhuT	SBP	Haem	AAC13287	*Pseudomonas aeruginosa* PAO1	–	–	–	6
ShuT	SBP	Haem	O70018	*Shigella dysenteriae*	–	–	–	7
FptX	Transporter	Pyochelin	AAG07606	*Pseudomonas aeruginosa* PAO1	SLG_13630	22	AmpG	8
RhtX	Transporter	Rhizobactin 1021	Q92XI7	*Sinorhizobium meliloti* 2011	–	–	–	8
FeoB	Transporter	Fe^{2+}	P33650	*Escherichia coli* K-12	SLG_36840	29	FeoB	9
			AAG07746	*Pseudomonas aeruginosa* PAO1	SLG_36840	28	FeoB	10
					SLG_p-00340	11	High-affinity iron transporter	
EfeU	Transporter	Fe^{2}	P75901	*Escherichia coli* K-12	–	–	–	11
MntH	Transporter	Mn^{2+}, Fe^{2+}	P0A769	*Escherichia coli* K-12	–	–	–	12
ZupT	Transporter	Zn^{2+}, Co^{2+}, Fe^{2+}	P0A8H3	*Escherichia coli* K-12	SLG_06990	31	ZIP family transporter	13
FutA1	SBP	Fe^{2+}	P72827	*Synechocystis* sp. strain PCC 6803	–	–	–	14
FutA2	SBP	Fe^{2+}	Q55835	*Synechocystis* sp. strain PCC 6803	–	–	–	15
YfeA	SBP	Fe^{2+}	Q56952	*Yersinia pestis*	–	–	–	16
**Outer membrane**								
FhuA	TBDR	Ferrichrome	P06971	*Escherichia coli* K-12	SLG_17010	26	TBDR	17
FhuE	TBDR	Coprogen	P16869	*Escherichia coli* K-12	SLG_04340	22	TBDR	18
Fiu	TBDR	Catecholate siderophore	P75780	*Escherichia coli* K-12	SLG_34550	30	TBDR	19
FoxA	TBDR	Ferrioxamine	Q9I116	*Pseudomonas aeruginosa* PAO1	SLG_17010	23	TBDR	20
FpvA	TBDR	Pyoverdine	P48632	*Pseudomonas aeruginosa* PAO1	SLG_10860	21	TBDR	21
FptA	TBDR	Pyochelin	P42512	*Pseudomonas aeruginosa* PAO1	SLG_17010	27	TBDR	22
PupA	TBDR	Pseudobactin 358	P25184	*Pseudomonas putida* WCS358	SLG_34550	20	TBDR	23
FcuA	TBDR	Ferrichrome	Q05202	*Yersinia enterocolitica*	SLG_04340	34	TBDR	24
TbpA	TBDR	Transferrin	AHW76545	*Neisseria meningitidis*	SLG_04380	22	TBDR	25
ShuA	TBDR	Haem	P72412	*Shigella dysenteriae*	SLG_04380	25	TBDR	26
HutA	TBDR	Haem, Haemoglobin	ACL95742	*Caulobacter crescentus* NA1000	SLG_04380	47	TBDR	27
HasR	TBDR	Haemophore	Q79AD2	*Serratia marcescens*	SLG_04380	21	TBDR	28

[a]SBP, Substrate binding protein of ABC transporter; TBDR, TonB-dependent receptor.
[b]Amino acid sequence identity was calculated using the EMBOSS Needle pairwise alignment program.

Table S3. Proteins most similar to SYK-6 TonB2, FiuA, and FeoAB in selected Sphingomonad strains

Species and strain	Number of *tonB*	Number of TBDR	TonB2		FiuA		FeoA		FeoB	
			Accession no.	Sequence identity (%)[a]	Accession no.	Sequence identity (%)[a]	Accession no.	Sequence identity (%)[a]	Accession no.	Sequence identity (%)[a]
Blastomonas natatoria	3	55	PXW77659	20	PXW77661	49	PXW76439	34	PXW76438	69
Novosphingobium aromaticivorans DSM12444	3	76	ABD24466	26	ABD27715	28	ABD27286	41	ABD27285	63
Novosphingobium nitrogenifigens DSM 19370	4	76	EGD58708	34	EGD58710	28	EGD57881	38	EGD57882	62
Novosphingobium pentaromativorans US6-1	4	68	EHJ58335	35	EHJ60384	32	EHJ60269	34	EHJ60270	62
Novosphingobium sp. PP1Y	3	98	CCA92020	22	CCA92021	33	CCA92126	37	CCA92127	62
Sphingobium chlorophenolicum L-1	5	94	AEG50735	33	AEG50736	49	AEG49960	60	AEG49961	70
Sphingobium japonicum UT26S	4	69	BAI95867	38	BAI95866	49	BAI95131	54	BAI95132	72
Sphingobium yanoikuyae ATCC 51230	8	100	EKU76051	35	EKU72862	54	EKU74251	48	EKU74252	70
Sphingomonas wittichii RW1	3	153	ABQ70391	35	ABQ69571	51	ABQ68809	35	ABQ68810	68
Sphingopyxis alaskensis RB2256	3	39	ABF54651	27	ABF53800	26	ABF54691	32	ABF54692	68

[a]Amino acid sequence identity was calculated using the EMBOSS Needle pairwise alignment program.

Table S4. Strains and plasmids used in this study

Strains or plasmids	Relevant characteristic(s)[a]	Reference or source
Strains		
Sphingobium sp.		
SYK-6	Wild type; Nal^r Sm^r	29
SME096	SYK-6 derivative; ΔSLG_36940 (*tonB3*)	30
SME097	SYK-6 derivative; ΔSLG_34540 (*tonB2*)	30
SME151	SYK-6 derivative; ΔSLG_13630	This study
SME219	SYK-6 derivative; ΔSLG_04340	This study
SME220	SYK-6 derivative; ΔSLG_04380	This study
SME221	SYK-6 derivative; ΔSLG_10860	This study
SME225	SYK-6 derivative; ΔSLG_34550 (*fiuA*)	This study
SME257	SYK-6 derivative; ΔSLG_12500 12510 (*ligAB*)	31
SME290	SYK-6 derivative; ΔSLG_37490 (*tonB4*)	30
SME292	SYK-6 derivative; ΔSLG_01650 (*tonB5*)	30
SME293	SYK-6 derivative; ΔSLG_14690 (*tonB6*)	30
SME303	SYK-6 derivative; Δ*tonB3456*	30
SME304	SYK-6 derivative; Δ*tonB23456*	30
SME305	SYK-6 derivative; ΔSLG_04340 SLG_04380 *fiuA*	This study
SME306	SYK-6 derivative; ΔSLG_04340 *fiuA*	This study
SME307	SYK-6 derivative; ΔSLG_04380 *fiuA*	This study
SME309	SYK-6 derivative; ΔSLG_05570 (*fur2*)	This study
SME310	SYK-6 derivative; ΔSLG_36840 (*feoB*)	This study
SME311	SYK-6 derivative; ΔSLG_10860 *fiuA*	This study
SME312	SYK-6 derivative; Δ*ligAB fiuA*	This study
SME313	SYK-6 derivative; ΔSLG_04340 SLG_04380 SLG_10860 *fiuA*	This study
SME314	SYK-6 derivative; ΔSLG_p-00340	This study
SME315	SYK-6 derivative; ΔSLG_06990	This study
Escherichia coli		
HB101	*recA13 supE44 hsd20 ara-14 proA2 lacY1 galK2 rpsL20 xyl-5 mtl-1*	32
NEB 10-beta	*araD*139 Δ(*ara-leu*)7697 *fhuA lacX74 galK* (ϕ80 Δ*lacZ* Δ*M15*) *recA1 endA1 nupG rpsL* (Sm^r) Δ(*mrr-hsdRMS-mcrBC*)	New England Biolabs
BL21(DE3)	F$^-$ *ompT hsdSB*(r_B^- m_B^-) *gal dcm* (DE3); T7 RNA polymerase gene under control of the *lacUV5* promoter	33
Plasmids		
pRK2013	Tra$^+$ Mob$^+$ ColE1 replicon; Km^r	34
pJB861	RK2 *ori* broad-host-range expression vector; Km^r P_m *xylS*	35
pAK405	Plasmid for allelic exchange and markerless gene deletions in Sphingomonads; Km^r	36
pSEVA225	RK2 *ori lacZ* promoter probe broad host range vector; Km^r	37
pSEVA338	pBBR1 *ori* broad-host-range expression vector; Cm^r P_m *xylS*	37
pET-16b	Expression vector; T7 promoter, Amp^r	Novagen
pJB-fiuA	pJB861 with a 2.3-kb BamHI fragment carrying *fiuA*	This study
pS-tonB2	pSEVA338 with a 0.9-kb fragment carrying *tonB2*	This study
pJB-tonB2	pJB861 with a 0.9-kb NotI-SacI fragment carrying *tonB2* from pS-tonB2	This study
pJB-tonB1	pJB861 with a 0.7-kb NotI-SacI fragment carrying *tonB1*	30
pJB-feoB	pJB861 with a 1.9 -kb BamHI fragment carrying *feoB*	This study
pAK4320	pAK405 with a 1.9-kb deletion cassette carrying up- and downstream regions of SLG_04320	This study
pAK4380	pAK405 with a 2.0-kb deletion cassette carrying up- and downstream regions of SLG_04380	This study
pAKfur2	pAK405 with a 2.2-kb deletion cassette carrying up- and downstream regions of *fur2*	This study
pAK6990	pAK405 with a 2.2-kb deletion cassette carrying up- and downstream regions of SLG_06990	This study

pAK10860	pAK405 with a 2.0-kb deletion cassette carrying up- and downstream regions of SLG_10860	This study
pAK13630	pAK405 with a 2.2-kb deletion cassette carrying up- and downstream regions of SLG_13630	This study
pAKfur1	pAK405 with a 2.3-kb deletion cassette carrying up- and downstream regions of SLG_29410 (*fur1*)	This study
pAKfiuA	pAK405 with a 2.0-kb deletion cassette carrying up- and downstream regions of *fiuA*	This study
pAKfeoB	pAK405 with a 2.2-kb deletion cassette carrying up- and downstream regions of *feoB*	This study
pAKp-00340	pAK405 with a 2.2-kb deletion cassette carrying up- and downstream regions of SLG_p-00340	This study
pS-4340	pSEVA225 with a 0.2-kb PCR amplicon carrying SLG_04340 promoter region	This study
pS-4380	pSEVA225 with a 0.4-kb PCR amplicon carrying SLG_04380 promoter region	This study
pS-10860	pSEVA225 with a 0.3-kb PCR amplicon carrying SLG_10860 promoter region	This study
pS-17010	pSEVA225 with a 0.2-kb PCR amplicon carrying SLG_17010 promoter region	This study
pS-t2	pSEVA225 with a 0.4-kb PCR amplicon carrying *tonB2* promoter region	This study
pS-fiuA	pSEVA225 with a 0.2-kb PCR amplicon carrying *fiuA* promoter region	This study
pS-t2-fiuA	pS-t2 with a 0.2-kb HindIII fragment carrying *fiuA* promoter region	This study
pS-feoA	pSEVA225 with a 0.1-kb PCR amplicon carrying *feoA* promoter region	This study
pS-t1	pSEVA225 with a 0.2-kb PCR amplicon carrying *tonB1* promoter region	This study
pET-fur1	pET-16b with a 0.4-kb NdeI-BamHI fragment carrying *fur1*	This study

[a] Nal^r, Sm^r, Km^r, Cm^r, and Amp^r, resistance to nalidixic acid, streptomycin, kanamycin, chloramphenicol, and ampicillin, respectively.

Table S5. Primers used in this study

Target gene	Primer	Sequences (5′ to 3′)
For gene disruption		
pAK4340	Dis_TopF	CGGTACCCGGGGATCGATGGGCTTGCTGTTCCTC
	Dis_TopR	GAAGCAGGGTCGGGAAAC
	Dis_BotF	GTTTCCCGACCCTGCTTCCCTTCGATGCGTTCAACCA
	Dis_BotR	CGACTCTAGAGGATCCTCCAGCTCCTCGGCATAA
pAK4380	Dis_TopF	CGGTACCCGGGGATCACATCATCGCCCAGCTCG
	Dis_TopR	TATGCGTGGTGGAGCGAC
	Dis_BotF	GTCGCTCCACCACGCATAAGAGCCCGTCATTGTCCT
	Dis_BotR	CGACTCTAGAGGATCGCCAAGAGCATCTGCAAGA
pAK5570	Dis_TopF	CGGTACCCGGGGATGTCCTCCGGCGTGTTGAA
(*fur2*)	Dis_TopR	GAACGCCCCGTGATCGAA
	Dis_BotF	TTCGATCACGGGGCGTTCGCCCTTGCCTTCAAGCGT
	Dis_BotR	CGACTCTAGAGGATCGTGAGGAGCCGCGTCATT
pAK6990	Dis_TopF	ATTCGAGCTCGGTACCCGGGAAGCCGGGCCAAGATCGT
	Dis_TopR	GTCGGGGATGATGATGTTGTCCCTCGCTGG
	Dis_BotF	ACAACATCATCATCCCCGACATGCATGAC
	Dis_BotR	CCTGCAGGTCGACTCTAGAGCATCTTTTCGCCACCACG
pAK10860	Dis_TopF	CGGTACCCGGGGATCGATGACTTCTGCGGCTGTA
	Dis_TopR	AGCGATGACCATAAACGAGT
	Dis_BotF	ACTCGTTTATGGTCATCGCTCATGGGCAACCTCAACAACC
	Dis_BotR	CGACTCTAGAGGATCCATGCTGCGGCGATGAAC
pAK13630	Dis_TopF	CGGTACCCGGGGATCGAAAGCAGGCTTGTCGGTC
	Dis_TopR	GAGCAGGAAGGCGACGAA
	Dis_BotF	TTCGTCGCCTTCCTGCTCCTTCTGGGCCTTCACGGT
	Dis_BotR	CGACTCTAGAGGATCGTATCGGGTTCAGCCTTCG
pAK29410	Dis_TopF	CGGTACCCGGGGATAGCATGCAGGCCACCGAT
(*fur1*)	Dis_TopR	AATCACACGGCGCTGCTC
	Dis_BotF	GAGCAGCGCCGTGTGATTATCGCGAGCGCAAGGACT
	Dis_BotR	CGACTCTAGAGGATCTGCGGACCGACGACGATA
pAK34550	Dis_TopF	CGGTACCCGGGGATCCTTCATGCACCCCAGCTTC
(*fiuA*)	Dis_TopR	CGACACAGGAAAGGGCAAG
	Dis_BotF	CTTGCCCTTTCCTGTGTCGGCAGCTCAACGTGAAGAACT
	Dis_BotR	CGACTCTAGAGGATCTTGTCCGCCCTCATGTCT
pAK36840	Dis_TopF	CGGTACCCGGGGATGTCGTTGCCAGCCGTTTCG
(*feoB*)	Dis_TopR	TGCCTGGTTCGTCTTCGC
	Dis_BotF	GCGAAGACGAACCAGGCAGGCTTGCGGCGATGAGAA
	Dis_BotR	CGACTCTAGAGGATCAAACGCCGCCTTCGATGG
pAKp-00340	Dis_TopF	CGGTACCCGGGGATGCCAGTCTCTCCTCCACA
	Dis_TopR	TAGTCGAGCAGCCGCCAT
	Dis_BotF	ATGGCGGCTGCTCGACTAGCAACTCCTGATGGCGGT
	Dis_BotR	CGACTCTAGAGGATCTGGCGTCGGCATTGATAC
For confirmation of gene disruption		
SLG_04340	Conf_F	TGAATCCCCGATCCTGACC
SLG_04380	Conf_F	TGTGATCGAGCGGGCTTT
fur2	Conf_F	GCCTGCGCGAACACATTG
SLG_06990	Conf_F	CTACATGTCGCTGAACGCG
SLG_10860	Conf_F	CCACTCCAGATGAAGCATGT
SLG_13630	Conf_F	ATCATGCGCTCGATATCCCG
SLG_17010	Conf_F	TTGGCTTTCCCCCGATTG
fiuA	Conf_F	GGTTGGGGTATGAACGCA
feoB	Conf_F	TCATGCCGTCAGCACGAC

SLG_p-00340	Conf_F	GATCGGCTTGAGGGTGGC
For RT-PCR		
04320-04330	Forward	GCGAGCTGGCGGAAATGA
	Reverse	GAGTCGCCCAGGAAATCG
04330-04340	Forward	CGATTTCCTGGGCGACTC
	Reverse	GCATCGTGGTGTAGGTCC
04340-04350	Forward	AACAAGGCGCAGGGCATT
	Reverse	GCGGATAGTCAACAGAGCC
04350-04360	Forward	GGCTCTGTTGACTATCCGC
	Reverse	CGGTCGGCACATAATGGA
feoA-feoB	Forward	ACGCATGATCGTGGGGAT
	Reverse	GCAACGCGGCGAAACTCT
tonB2-fiuA	Forward	GTGGACCGCAGGATATGG
	Reverse	CACAGGAAAGGGCAAGGA
fiuA-34560	Forward	AGCATCACCGCCAACTAC
	Reverse	GGGAAGATCGCATTGTCG
34560-34570	Forward	CGACAATGCGATCTTCCC
	Reverse	GTCTGTTCGCGCGTTTCC
For EMSA		
SLG_04320	Forward (+Fur box)	TACCCGCTCGATTTGCCTC
	Forward (-Fur box)	ATGACGAGTGCGGGATCAG
	Reverse	GTAGAGCCCGTCATTGTCC
SLG_04380	Forward (+Fur box)	GTGAGGGCGTATGCAGAGG
	Forward (-Fur box)	GCGAACACTGAAGGTCCGT
	Reverse	TGGAAGATCAACGGCAGGC
tonB2	Forward (+Fur box)	CGGCTCCGAATATCTCTGC
	Forward (-Fur box)	TCGGTTGGGGTATGAACGC
	Reverse	GAAGCTGGGGTGCATGAAG
fiuA	Forward (+Fur box)	CGCATCCTCTACAGATCGG
	Forward (-Fur box)	CAACAGCAGACAGGGGAAC
	Reverse	GGTATCGGTGACGGTGAC
feoB	Forward (+Fur box)	GACTGCGGGTCGATAGCC
	Forward (-Fur box)	AGAAAGCTCCGTCTTGCG
	Reverse	ATCCCCACGATCATGCGT
For plasmid construction		
pJB-fiuA	Forward	GAAGCTTCGTGGATCTCCAGTCGAACAACCGTG
	Reverse	CAGGATATCTGGATCCATGAGCATTTCCTTCGCC
pS-tonB2	Forward	GCCTAGGCCGCGGCCGCGCGATTACAGGACCGGGCACG
	Reverse	ATCCCCGGGTACCGAGCTCGTCAGGTCTCGATCGTCTTG
pJB-feoB	Forward	GAAGCTTCGTGGATCTCGGCCTTGCCGCGTCAG
	Reverse	CAGGATATCTGGATCCTAGAGGCCCAGGGCCAC
pS-4320	Forward	CCTCTAGAGTCGACCGGAAACCTCCAGACCAGA
	Reverse	CTAAGCTTGCATGCCGGACCTTCAGTGTTCGCT
pS-4380	Forward	CCTCTAGAGTCGACCGAGCGTCGCGGCGCCGAT
	Reverse	CTAAGCTTGCATGCCGCGCGCGGTTCCACGGTC
pS-10860	Forward	CCTCTAGAGTCGACCGCTGTTAGCACTCACCCT
	Reverse	CTAAGCTTGCATGCCGAATAGCCCCCGAAAATGT
pS-17010	Forward	CCTCTAGAGTCGACCCCTGACGATCGAGGCGGC
	Reverse	CTAAGCTTGCATGCCGACAGCCCCCTCGGAAGA
pS-t2	Forward	CCTCTAGAGTCGACCCGGCGCCGGCGCAGAAGC
	Reverse	CTAAGCTTGCATGCCACCCCAACCGATCGTGCC

pS-fiuA	Forward	CCTCTAGAGTCGACCGCGCGGCCGGCGCGGGCA
	Reverse	CTAAGCTTGCATGCCTGGTTCCCCTGTCTGCTG
pS-t2-fiuA	Forward	ATCGGTTGGGGTGGCATGCAGCGCGGCCGGCGCGGGCA
	Reverse	GTCATATGTTTTTCCTCCTATGGTTCCCCTGTCTGCTGTTGTGCAT TTCGCCGACGC
pS-feoA	Forward	CCTCTAGAGTCGACCAAGGTCCGACTGCGGGTC
	Reverse	CTAAGCTTGCATGCCAAGACGGAGCTTTCTTCGA
pS-t1	Forward	CCTCTAGAGTCGACCTAAGATGATTCTCCGGAGG
	Reverse	CTAAGCTTGCATGCCTTCAGCAACGAAACTCCTTA
pET-fur1	Forward	GGCCATACGAAGGTCGTCATACCCGGACAATCGATATTGAAGC
	Reverse	TCGGGCTTTGTTAGCAGCCGTCAGTCCTTGCGCTCGCG

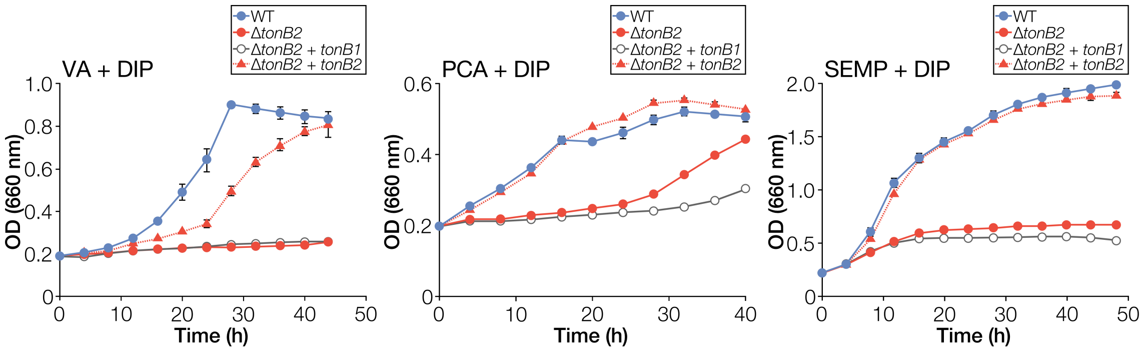

Fig. S1. Growth complementation of Δ*tonB2* under iron-limited conditions. Cells of SYK-6(pJB861, vector), Δ*tonB2*(pJB861), Δ*tonB2*(pJB-tonB1), and Δ*tonB2*(pJB-tonB2) were cultured in Wx medium containing 5 mM VA, 5 mM PCA, or SEMP with 100 µM DIP and 1 mM *m*-toluate. Cell growth was monitored by measuring the OD_{660}. Each value is the average ± the standard deviation of three independent experiments.

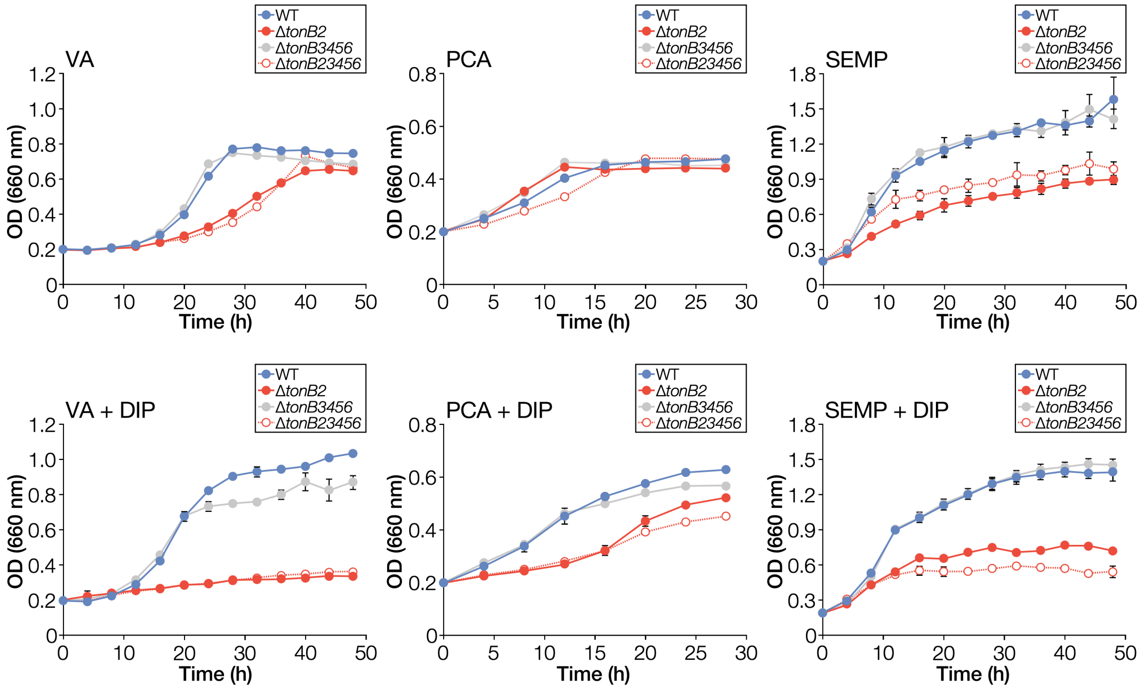

Fig. S2. Growth of *tonB* multiple mutants on VA, PCA, and SEMP. Cells of SYK-6, Δ*tonB2*, Δ*tonB3456*, and Δ*tonB23456* were cultured in Wx medium containing 5 mM VA, 5 mM PCA, or SEMP in the presence or absence of 100 µM DIP. Cell growth was monitored by measuring the OD_{660}. Each value is the average ± the standard deviation of three independent experiments.

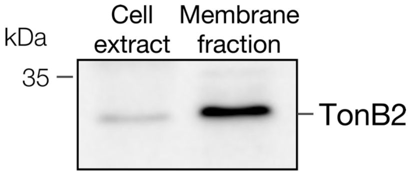

Fig. S3. Cellular localisation of TonB2. Western blot analysis using anti-TonB2 antibodies was performed against cell extract and total membrane fraction (10 µg protein) obtained from SYK-6 cells grown in LB. The uncropped blot image is shown in Fig. S21.

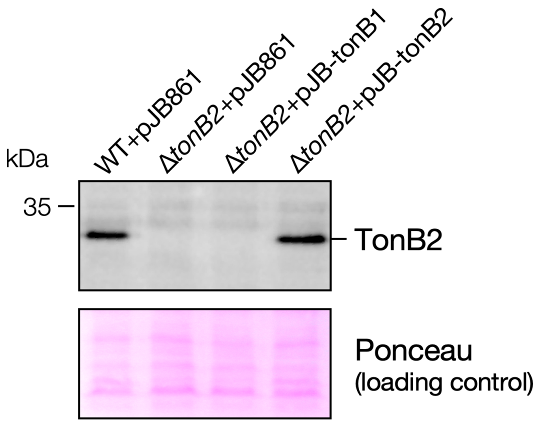

Fig. S4. Expression of *tonB2* in a *tonB2*-complemented Δ*tonB2*. Western blot analysis using anti-TonB2 antibodies was performed against total membrane fractions (10 µg protein) obtained from SYK-6(pJB861, vector), Δ*tonB2*(pJB861), Δ*tonB2*(pJB-tonB1), and Δ*tonB2*(pJB-tonB2) cells grown in LB containing 1 mM *m*-toluate. Ponceau staining is shown as loading control. The uncropped blot and ponceau staining image are shown in Fig. S21.

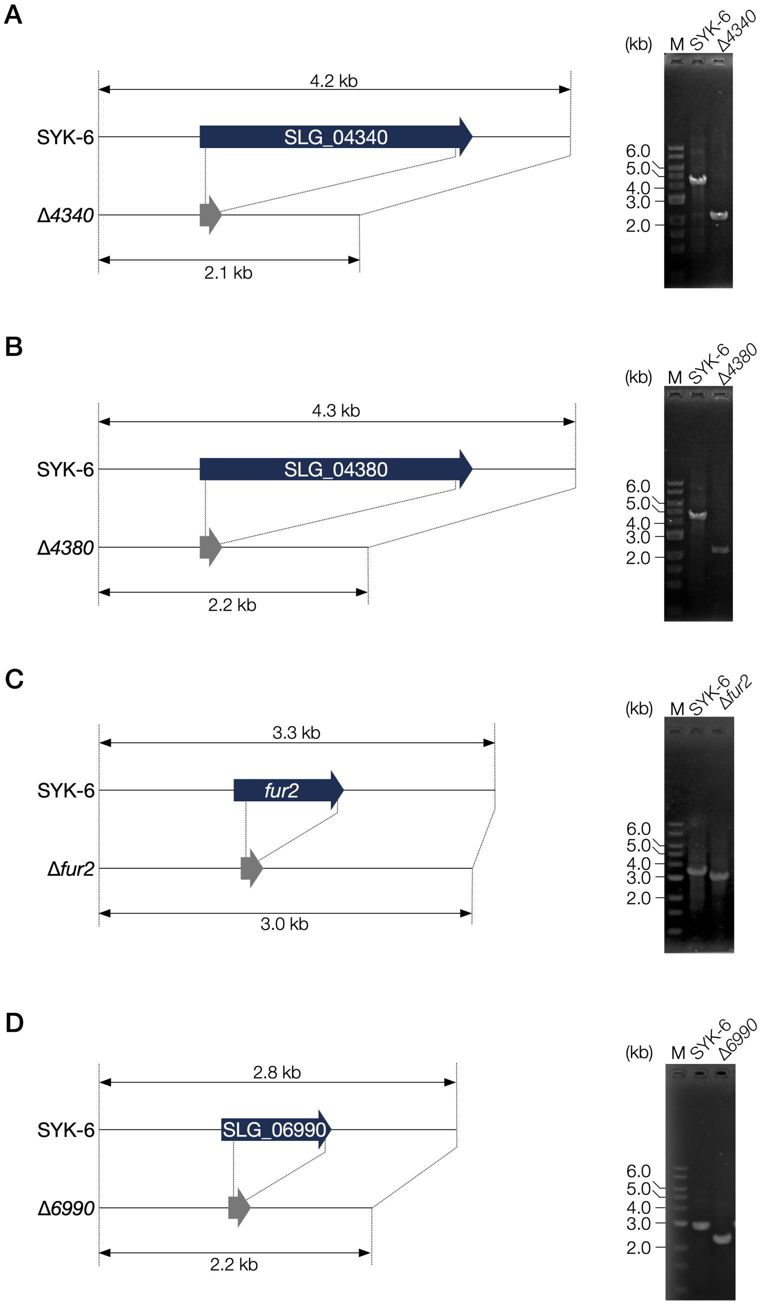

Fig. S5. Construction of mutants. The panels on the left show schematic representations of the disruption of SLG_04340 (A), SLG_04380 (B), *fur2* (C), SLG_06990 (D), SLG_10860 (E), SLG_13630 (F), *fiuA* (G), *feoB* (H), and SLG_p-00340 (I). The disruption of the genes was examined by colony PCR analyses. The primer pairs used for colony PCR analyses are shown in Table S5 (Conf_F and Dis_BotR). M, molecular size markers.

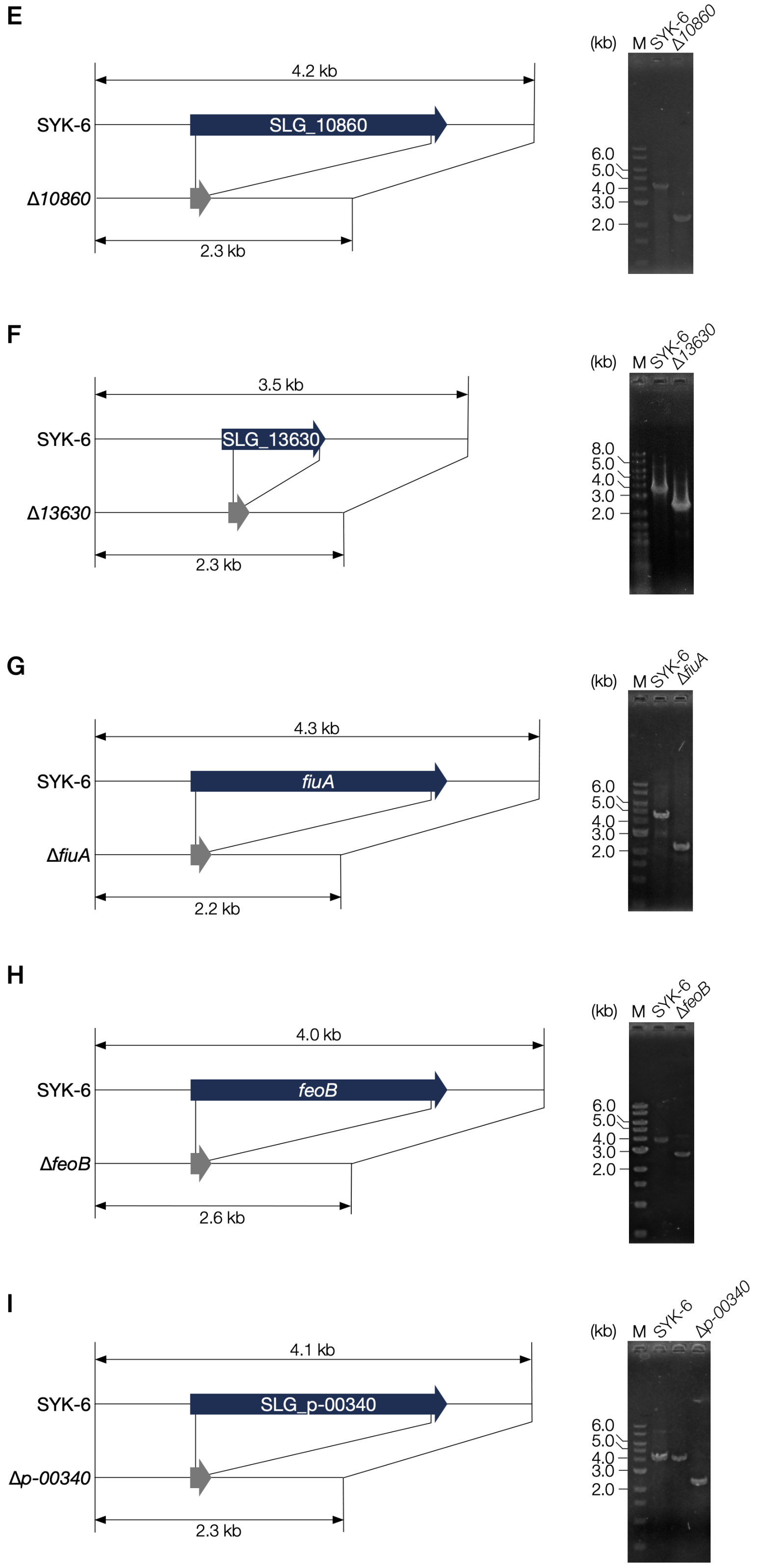

Fig. S5. *–continued.*

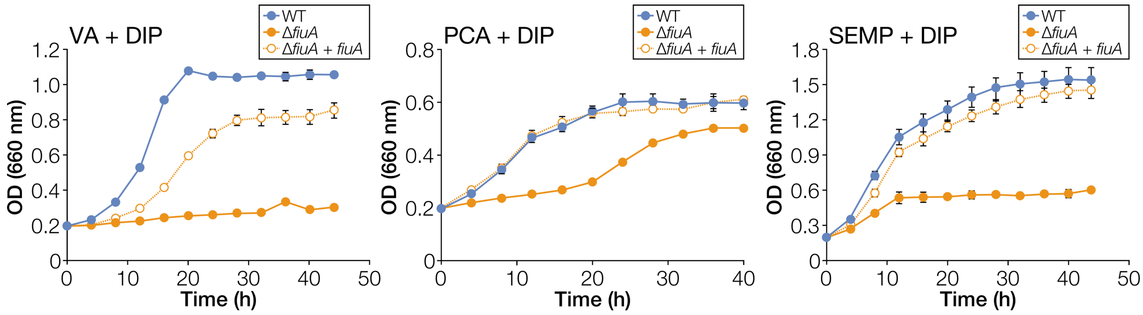

Fig. S6. Growth complementation of Δ*fiuA* under iron-limited conditions. Cells of SYK-6(pJB861, vector), Δ*fiuA*(pJB861), and Δ*fiuA*(pJB-fiuA) were cultured in Wx medium containing 5 mM VA, 5 mM PCA, or SEMP with 100 µM DIP and 1 mM *m*-toluate. Cell growth was monitored by measuring the OD_{660}. Each value is the average ± the standard deviation of three independent experiments.

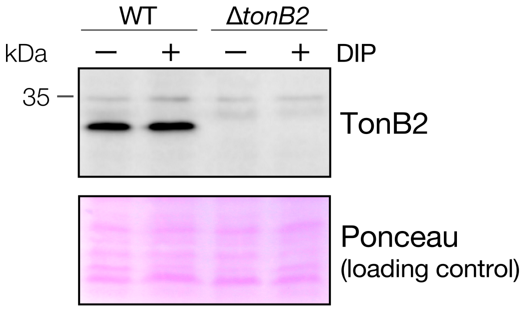

Fig. S7. Level of TonB2 production under iron-limited conditions. Western blot analysis using anti-TonB2 antibodies was performed against total membrane fractions (10 μg protein) obtained from SYK-6 and Δ*tonB2* cells grown in LB with or without 100 μM DIP. The band intensities of TonB2 determined by LumiVision PRO image analyser (Aisin Seiki Co., Ltd) were 222,851,628 (WT − DIP) and 258,797,925 (WT + DIP), respectively. The uncropped blot and ponceau staining image are shown in Fig. S21.

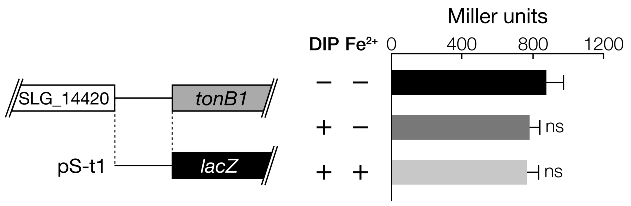

Fig. S8. Promoter activities of *tonB1* under iron-replete and limited conditions. β-galactosidase activities of SYK-6 cells harbouring pS-t1 grown in Wx-SEMP with or without 100 µM DIP and 100 µM $FeCl_2$ are shown. The DNA fragments used for the promoter analysis are shown on the left. Each value is the average ± the standard deviation of three independent experiments. ns, $P > 0.05$ (one-way ANOVA with Dunnett's multiple comparisons).

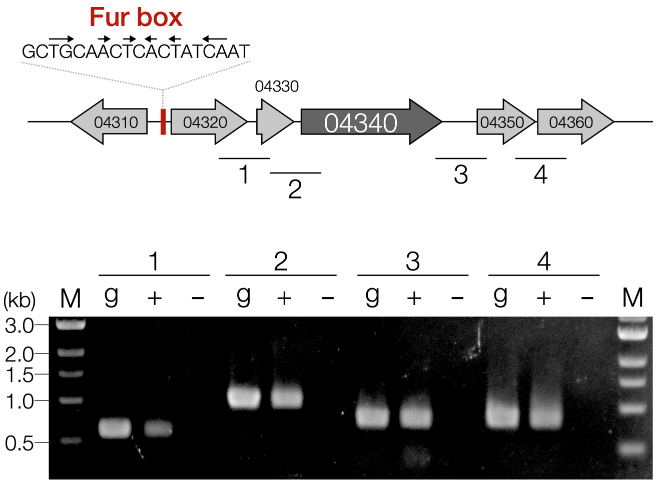

Fig. S9. RT-PCR analysis of the SLG_04320–04360 gene cluster. Total RNA used for cDNA synthesis was isolated from SYK-6 cells grown in Wx-SEMP with 100 μM DIP. The regions to be amplified are indicated by black bars below the genetic map. Lanes: M, molecular size markers; g, control PCR with the SYK-6 genomic DNA; '+' and '–', RT-PCR with and without reverse transcriptase, respectively.

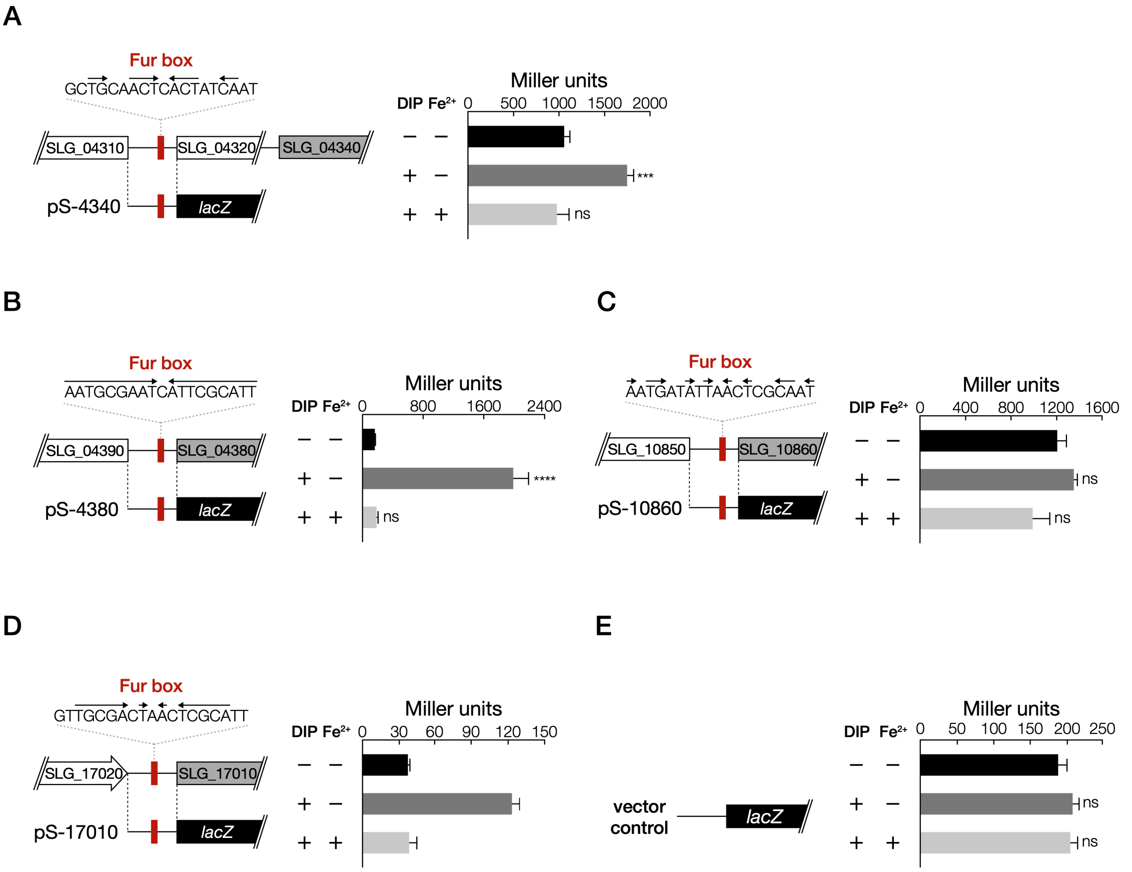

Fig. S10. Promoter activities of TBDR candidate genes involved in iron uptake under iron-replete and limited conditions. β-galactosidase activities of SYK-6 cells harbouring pS-4340 (A), pS-4380 (B), pS-10860 (C), pS-17010 (D), or pSEVA225 (E) grown in Wx-SEMP with or without 100 µM DIP and 100 µM $FeCl_2$ are shown. The DNA fragments used for the promoter analysis are shown on the left. Each value is the average ± the standard deviation of three independent experiments. ns, $P > 0.05$, ***, $P < 0.001$, ****, $P < 0.0001$ (one-way ANOVA with Dunnett's multiple comparisons).

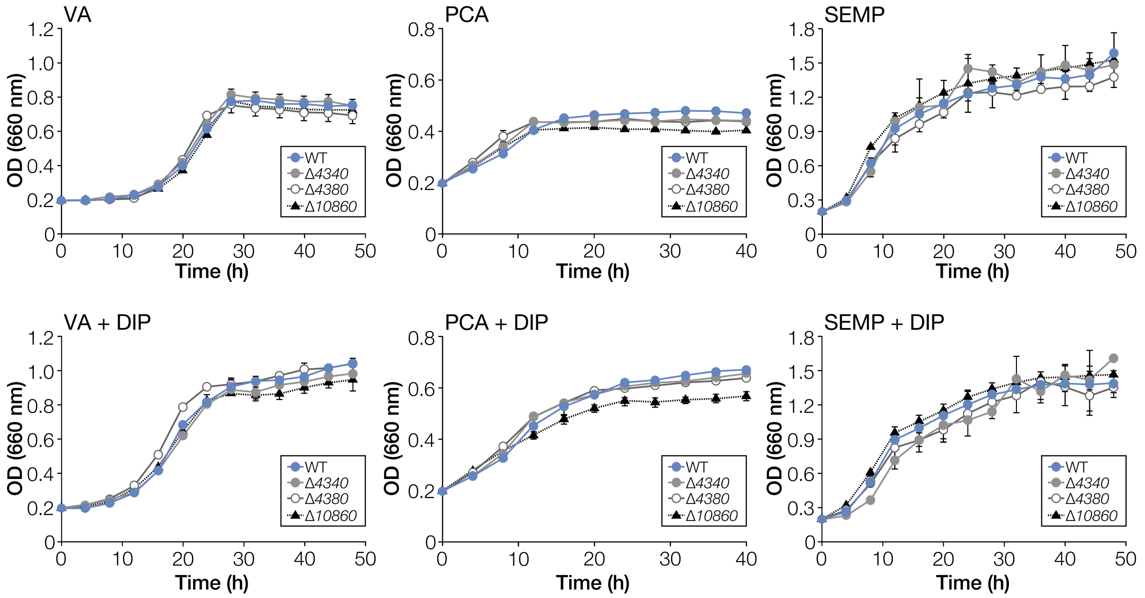

Fig. S11. Growth of Δ*4340*, Δ*4380*, and Δ*10860* on VA, PCA, and SEMP. Cells of SYK-6, Δ*4340*, Δ*4380*, and Δ*10860* were cultured in Wx medium containing 5 mM VA, 5 mM PCA, or SEMP in the presence or absence of 100 µM DIP. Cell growth was monitored by measuring the OD_{660}. Each value is the average ± the standard deviation of three independent experiments.

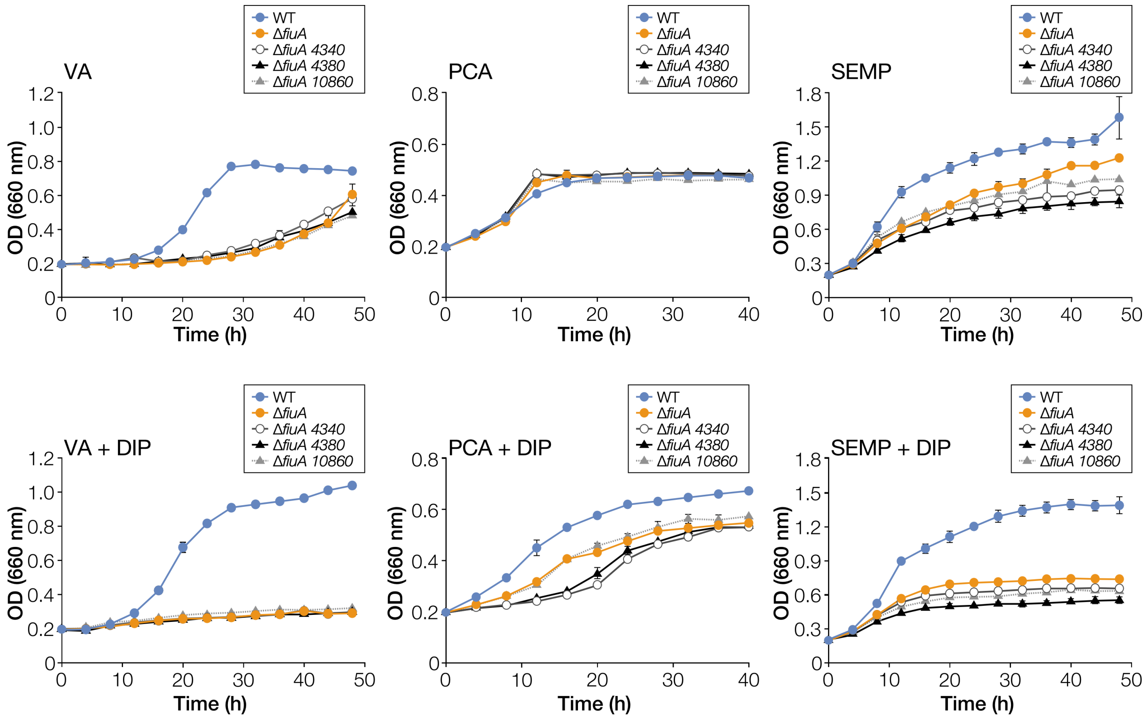

Fig. S12. Growth of Δ*fiuA 4340*, Δ*fiuA 4380*, and Δ*fiuA 10860* on VA, PCA, and SEMP. Cells of SYK-6, Δ*fiuA*, Δ*fiuA 4340*, Δ*fiuA 4380*, and Δ*fiuA 10860* were cultured in Wx medium containing 5 mM VA, 5 mM PCA, or SEMP in the presence or absence of 100 μM DIP. Cell growth was monitored by measuring the OD_{660}. Each value is the average ± the standard deviation of three independent experiments.

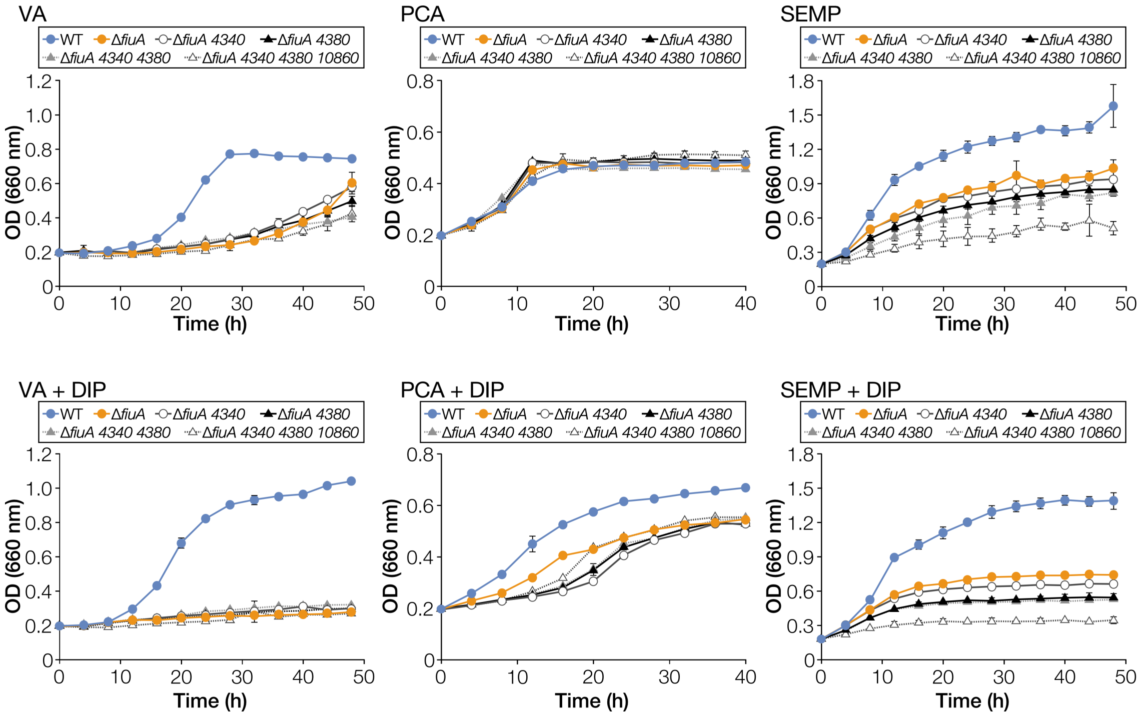

Fig. S13. Growth of Δ*fiuA 4340 4380* and Δ*fiuA 4340 4380 10860* on VA, PCA, and SEMP. Cells of SYK-6, Δ*fiuA*, Δ*fiuA 4340*, Δ*fiuA 4380*, Δ*fiuA 4340 4380*, and Δ*fiuA 4340 4380 10860* were cultured in Wx medium containing 5 mM VA, 5 mM PCA, or SEMP in the presence or absence of 100 µM DIP. Cell growth was monitored by measuring the OD_{660}. Each value is the average ± the standard deviation of three independent experiments.

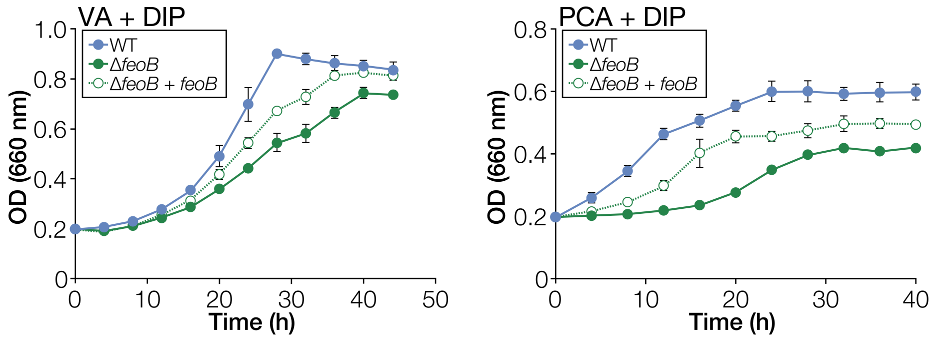

Fig. S14. Growth complementation of Δ*feoB* under iron-limited conditions. Cells of SYK-6(pJB861, vector), Δ*feoB*(pJB861), and Δ*feoB*(pJB-feoB) were cultured in Wx medium containing 5 mM VA or 5 mM PCA with 100 µM DIP and 1 mM *m*-toluate. Cell growth was monitored by measuring the OD_{660}. Each value is the average ± the standard deviation of three independent experiments.

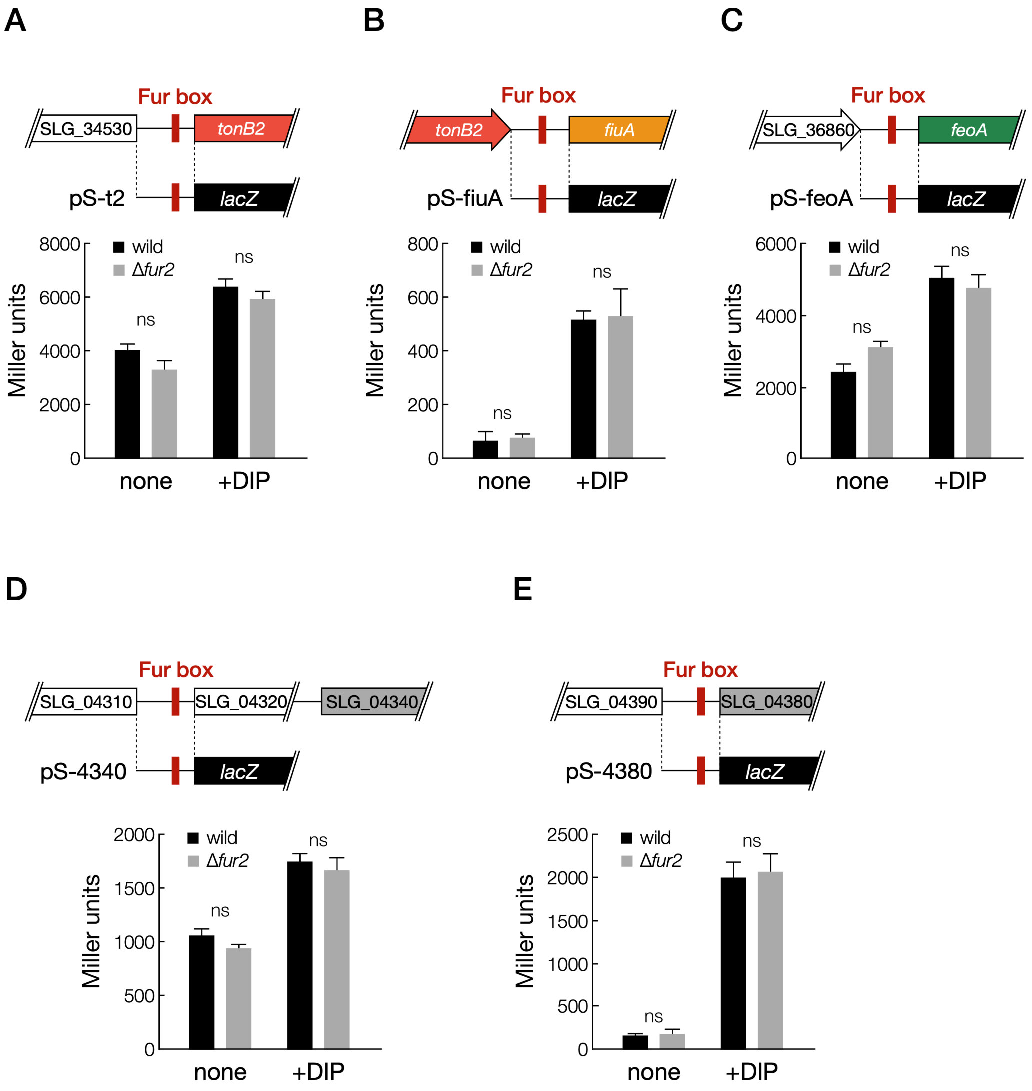

Fig. S15. Disruption of *fur2* did not affect the promoter activities of iron transporter genes. β-galactosidase activities of SYK-6 and Δ*fur2* cells harbouring pS-t2 (A), pS-fiuA (B), pS-feoA (C), pS-4340 (D), or pS-4380 (E) grown in Wx-SEMP with or without 100 μM DIP are shown. The DNA fragments used for the promoter analysis are shown at the top. Each value is the average ± the standard deviation of three independent experiments. ns, $P > 0.05$ (two-tailed, unpaired t-test).

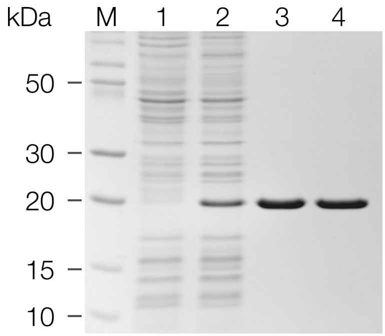

Fig. S16. Purification of Fur1. Proteins were separated using SDS-15% PAGE and stained with Coomassie Brilliant Blue. Lanes: 1, cell extracts of *E. coli* BL21(DE3) harbouring pET-16b (10 μg protein); 2, cell extracts of *E. coli* BL21(DE3) harbouring pET-fur1 (10 μg protein); 3, Fur1 purified by His Spin Trap (2.0 μg protein); 4, purified Fur1 after ultrafiltration (2.0 μg protein); M, molecular size markers.

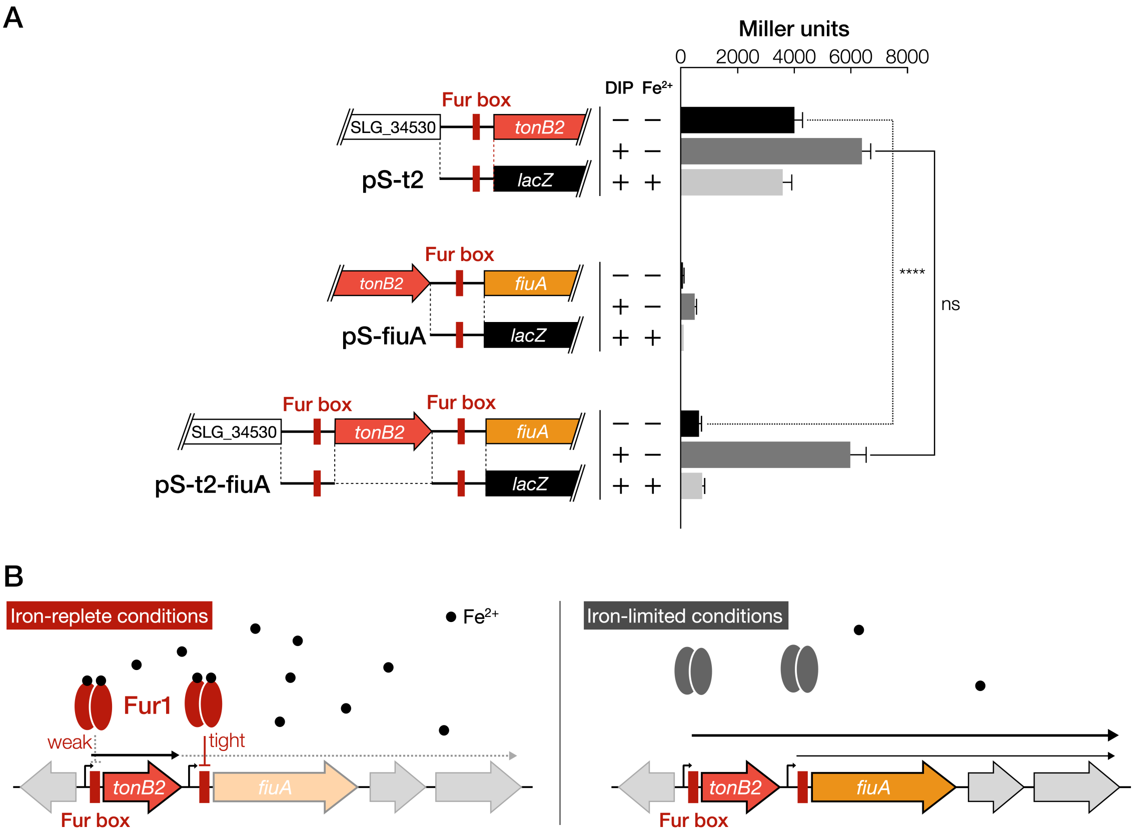

Fig. S17. Transcription of *fiuA* is tightly regulated by Fur1. (**A**) β-galactosidase activities of SYK-6 cells harbouring pS-t2, pS-fiuA, or pS-t2-fiuA grown in Wx-SEMP with or without 100 µM DIP and 100 µM $FeCl_2$. The DNA fragments used for the promoter analysis are shown on the left. Each value is the average ± the standard deviation of three independent experiments. ns, $P > 0.05$, ****, $P < 0.0001$ (two-tailed, unpaired *t*-test). (**B**) Proposed transcriptional regulation of the *tonB2-fiuA* operon. Under iron-replete conditions, transcription of *fiuA* from the *tonB2* promoter is interrupted by binding of Fur1 to the Fur box of *fiuA* (left). Under iron-limited conditions, Fur1 is released from the Fur boxes, and *fiuA* is strongly transcribed from the *tonB2* promoter (right).

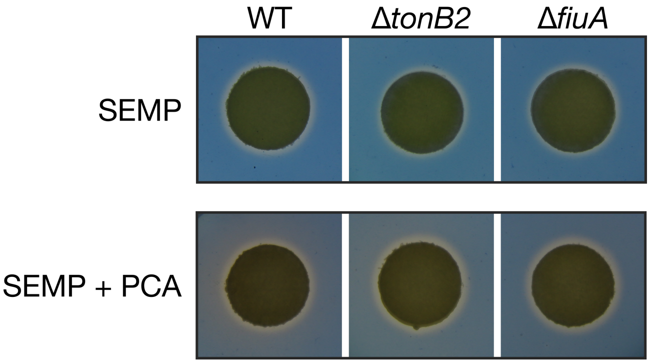

Fig. S18. Production of siderophores by SYK-6, Δ*tonB2*, and Δ*fiuA*. Cells of SYK-6, Δ*tonB2*, and Δ*fiuA* were incubated on Wx-SEMP-CAS agar plates with or without 1 mM PCA for 144 h.

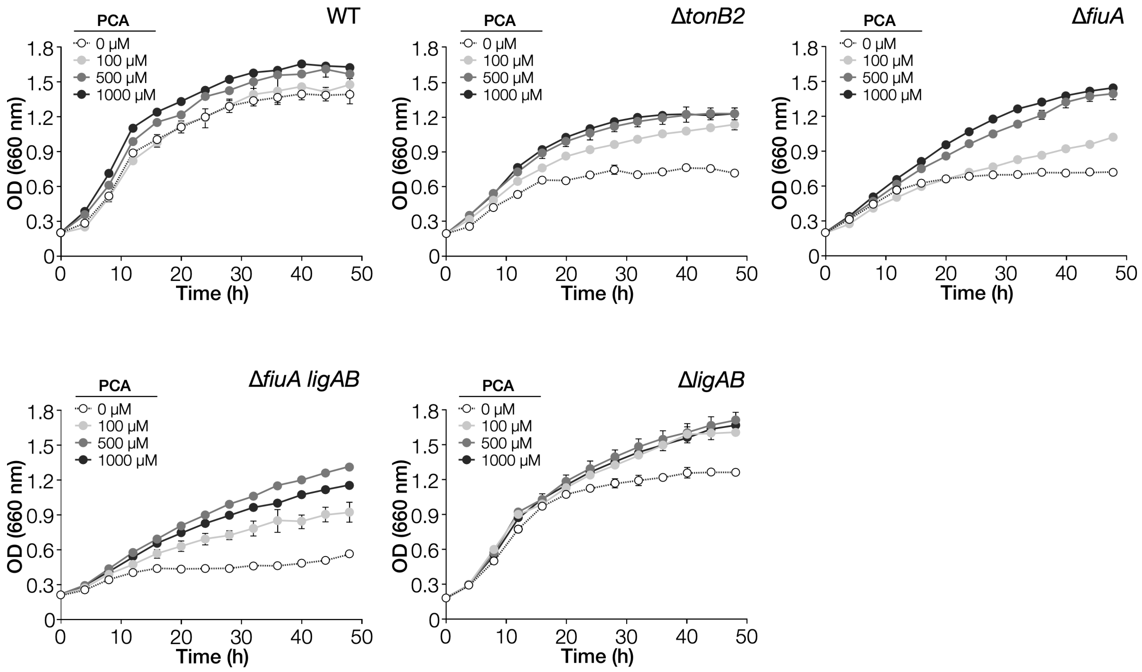

Fig. S19. Effect of PCA addition on the growth of Δ*tonB2* and Δ*fiuA* on SEMP under iron-limited conditions. Cells of SYK-6, Δ*tonB2*, Δ*fiuA*, Δ*fiuA ligAB*, and Δ*ligAB* were cultured in Wx-SEMP containing 100 µM DIP with or without PCA (100 µM, 500 µM, or 1000 µM). Cell growth was monitored by measuring the OD_{660}. Each value is the average ± the standard deviation of three independent experiments.

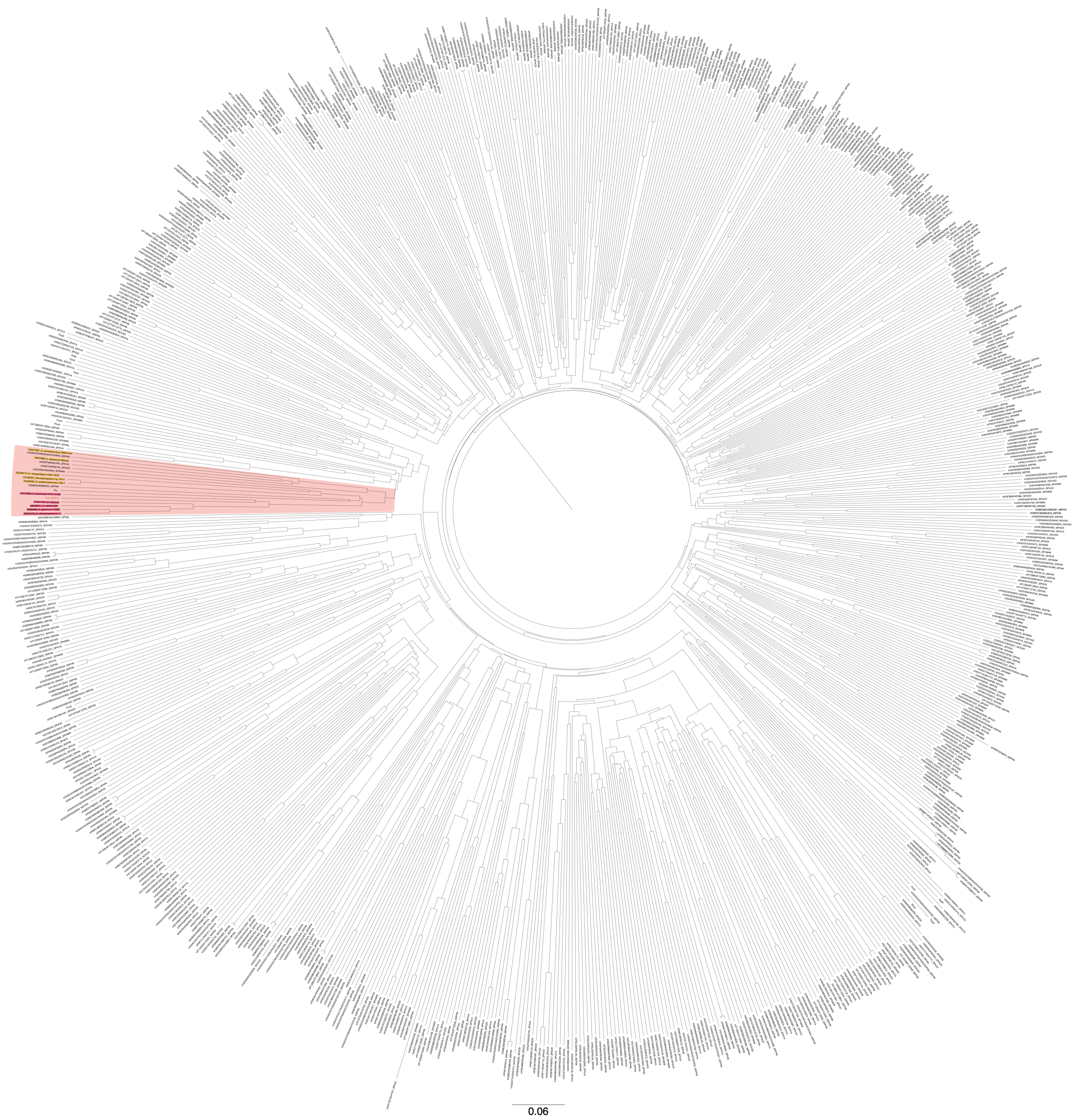

Fig. S20. Phylogenetic tree of Sphingomonad TBDRs with known iron-uptake TBDRs. TBDRs classified into the same clade with *fiuA* are highlighted with a red background. Among TBDRs listed in Table S3, TBDRs highlighted in magenta and yellow show >40% and 26–33% amino acid sequence identities with *fiuA*, respectively. The accession numbers of TBDR are shown in the figure, Table S2, and S3. The scale bar corresponds to 0.06 amino acid substitutions per position. A multiple alignment was performed using the Clustal Omega program[38].

A

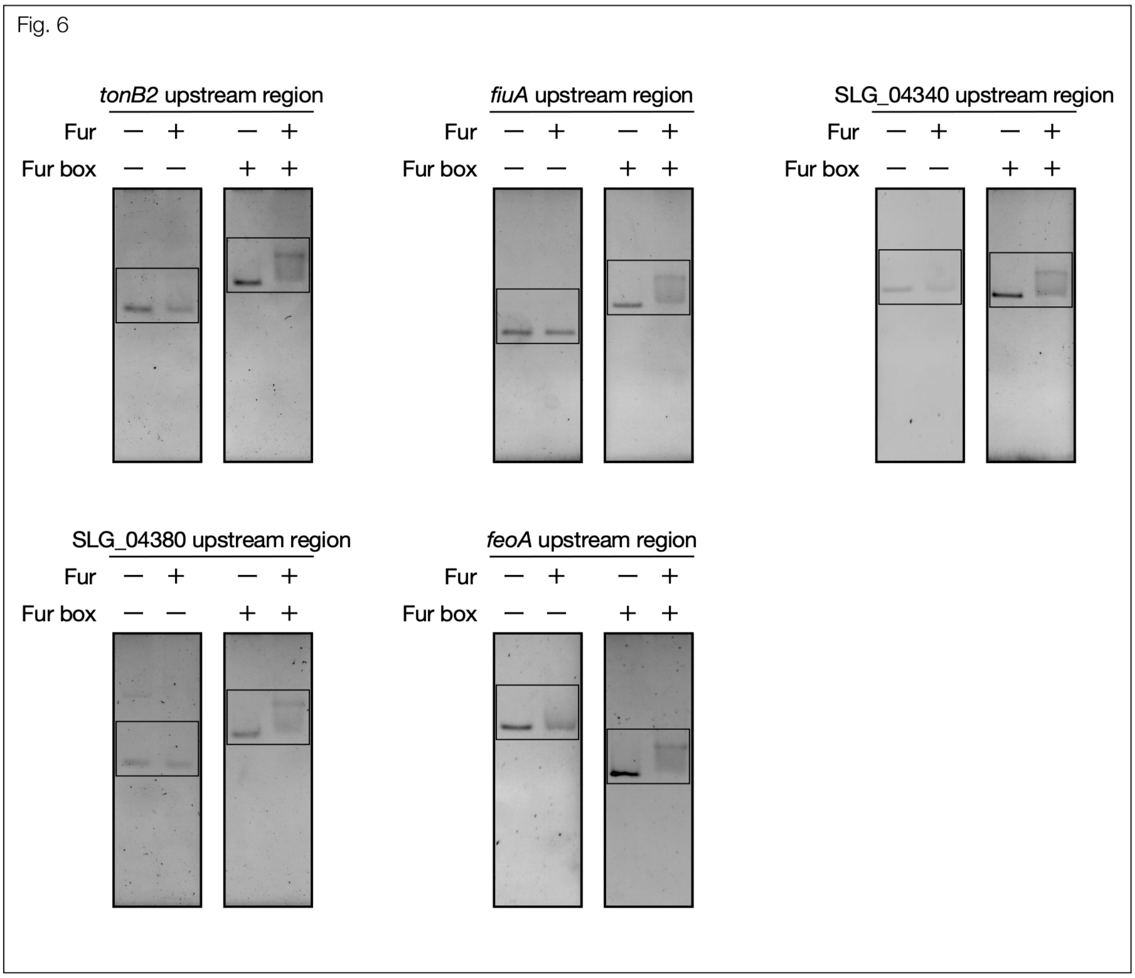

B

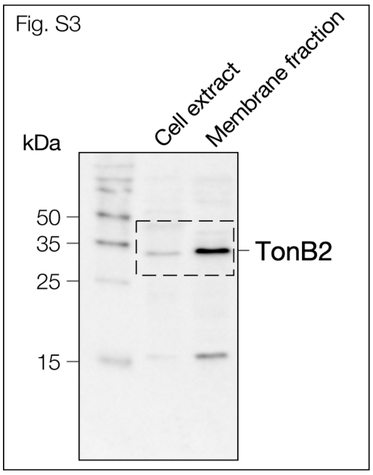

Fig. S21. Uncropped western blots, ponceau staining, and EMSA images shown in Fig. 6, Fig. S3, Fig. S4, and Fig. S7.

C

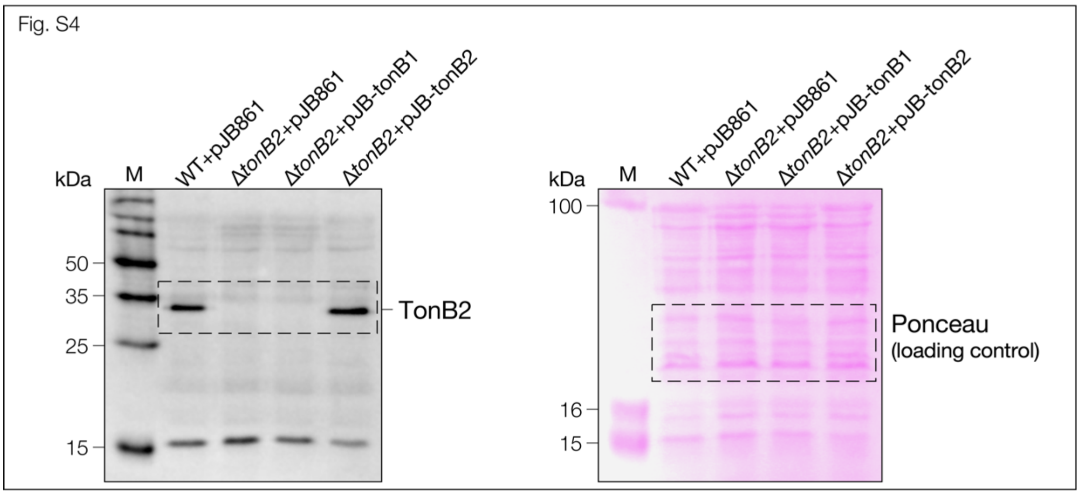

D

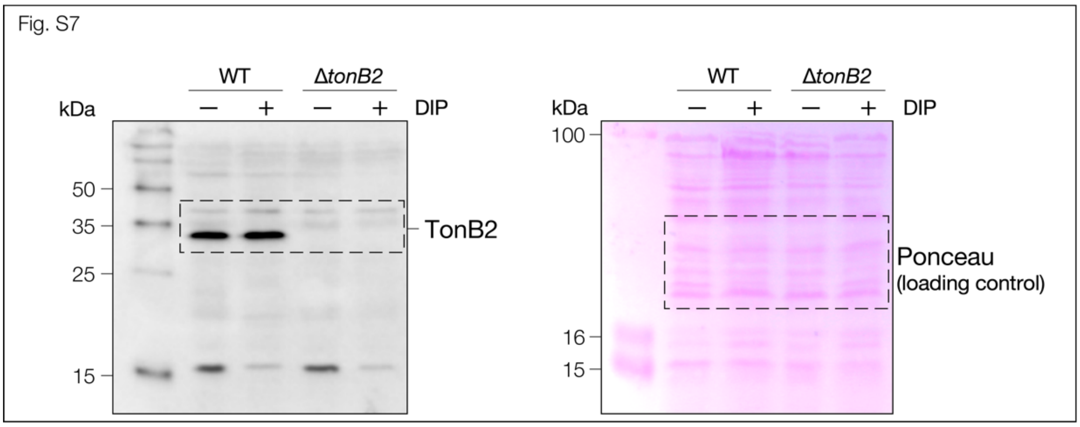

Fig. S21. –*continued.*

Supplementary References

1 Stephens, D. L., Choe, M. D. & Earhart, C. F. *Escherichia coli* periplasmic protein FepB binds ferrienterobactin. *Microbiology* **141**, 1647-1654, doi:10.1099/13500872-141-7-1647 (1995).

2 Clarke, T. E., Rohrbach, M. R., Tari, L. W., Vogel, H. J. & Köster, W. Ferric hydroxamate binding protein FhuD from *Escherichia coli*: mutants in conserved and non-conserved regions. *Biometals* **15**, 121-131, doi:10.1023/A:1015249530156 (2002).

3 Staudenmaier, H., Van Hove, B., Yaraghi, Z. & Braun, V. Nucleotide sequences of the *fecBCDE* genes and locations of the proteins suggest a periplasmic-binding-protein-dependent transport mechanism for iron(III) dicitrate in *Escherichia coli*. *J. Bacteriol.* **171**, 2626-2633, doi:10.1128/jb.171.5.2626-2633.1989 (1989).

4 Grigg, J. C., Cheung, J., Heinrichs, D. E. & Murphy, M. E. Specificity of staphyloferrin B recognition by the SirA receptor from *Staphylococcus aureus*. *J. Biol. Chem.* **285**, 34579-34588, doi:10.1074/jbc.M110.172924 (2010).

5 Naka, H., López, C. S. & Crosa, J. H. Role of the pJM1 plasmid-encoded transport proteins FatB, C and D in ferric anguibactin uptake in the fish pathogen *Vibrio anguillarum*. *Environ. Microbiol. Rep.* **2**, 104-111, doi:10.1111/j.1758-2229.2009.00110.x (2010).

6 Ho, W. W. *et al.* Holo- and apo-bound structures of bacterial periplasmic heme-binding proteins. *J. Biol. Chem.* **282**, 35796-35802, doi:10.1074/jbc.M706761200 (2007).

7 Eakanunkul, S. *et al.* Characterization of the periplasmic heme-binding protein shut from the heme uptake system of *Shigella dysenteriae*. *Biochemistry* **44**, 13179-13191, doi:10.1021/bi050422r (2005).

8 Cuív, P. O., Clarke, P., Lynch, D. & O'Connell, M. Identification of *rhtX* and *fptX*, novel genes encoding proteins that show homology and function in the utilization of the siderophores rhizobactin 1021 by *Sinorhizobium meliloti* and pyochelin by *Pseudomonas aeruginosa*, respectively. *J. Bacteriol.* **186**, 2996-3005, doi:10.1128/jb.186.10.2996-3005.2004 (2004).

9 Kammler, M., Schön, C. & Hantke, K. Characterization of the ferrous iron uptake system of *Escherichia coli*. *J. Bacteriol.* **175**, 6212-6219, doi:10.1128/jb.175.19.6212-6219.1993 (1993).

10 Marshall, B., Stintzi, A., Gilmour, C., Meyer, J. M. & Poole, K. Citrate-mediated iron uptake in *Pseudomonas aeruginosa*: involvement of the citrate-inducible FecA receptor and the FeoB ferrous iron transporter. *Microbiology* **155**, 305-315, doi:10.1099/mic.0.023531-0 (2009).

11 Grosse, C. *et al.* A new ferrous iron-uptake transporter, EfeU (YcdN), from *Escherichia coli*. *Mol. Microbiol.* **62**, 120-131, doi:10.1111/j.1365-2958.2006.05326.x (2006).

12 Makui, H. *et al.* Identification of the *Escherichia coli* K-12 Nramp orthologue (MntH) as a selective divalent metal ion transporter. *Mol. Microbiol.* **35**, 1065-1078, doi:10.1046/j.1365-2958.2000.01774.x (2000).

13 Grass, G. *et al.* The metal permease ZupT from *Escherichia coli* is a transporter with a broad substrate spectrum. *J. Bacteriol.* **187**, 1604-1611, doi:10.1128/JB.187.5.1604-1611.2005 (2005).

14 Katoh, H., Hagino, N. & Ogawa, T. Iron-binding activity of FutA1 subunit of an ABC-type iron transporter in the cyanobacterium *Synechocystis* sp. Strain PCC 6803. *Plant Cell Physiol.* **42**, 823-827, doi:10.1093/pcp/pce106 (2001).

15 Badarau, A. *et al.* FutA2 is a ferric binding protein from *Synechocystis* PCC 6803. *J. Biol. Chem.* **283**, 12520-12527, doi:10.1074/jbc.M709907200 (2008).

16 Perry, R. D., Mier, I. & Fetherston, J. D. Roles of the Yfe and Feo transporters of *Yersinia pestis* in iron uptake and intracellular growth. *BioMetals* **20**, 699, doi:10.1007/s10534-006-9051-x (2007).

17 Pawelek, P. D. *et al.* Structure of TonB in complex with FhuA, *E. coli* outer membrane receptor. *Science* **312**, 1399-1402, doi:10.1126/science.1128057 (2006).

18 Sauer, M., Hantke, K. & Braun, V. Ferric-coprogen receptor FhuE of *Escherichia coli*: processing and sequence common to all TonB-dependent outer membrane receptor proteins. *J. Bacteriol.* **169**, 2044-2049, doi:10.1128/jb.169.5.2044-2049.1987 (1987).

19 Nikaido, H. & Rosenberg, E. Y. Cir and Fiu proteins in the outer membrane of *Escherichia coli* catalyze transport of monomeric catechols: study with β-lactam antibiotics containing catechol and analogous groups. *J. Bacteriol.* **172**, 1361-1367, doi:10.1128/jb.172.3.1361-1367.1990 (1990).

20 Llamas, M. A. *et al.* The heterologous siderophores ferrioxamine B and ferrichrome activate signaling pathways in *Pseudomonas aeruginosa*. *J. Bacteriol.* **188**, 1882-1891, doi:10.1128/JB.188.5.1882-1891.2006 (2006).

21 Meyer, J. M., Stintzi, A. & Poole, K. The ferripyoverdine receptor FpvA of *Pseudomonas aeruginosa* PAO1 recognizes the ferripyoverdines of *P. aeruginosa* PAO1 and *P. fluorescens* ATCC 13525. *FEMS Microbiol. Lett.* **170**, 145-150, doi:10.1111/j.1574-6968.1999.tb13367.x (1999).

22 Ankenbauer, R. G. & Quan, H. N. FptA, the Fe(III)-pyochelin receptor of *Pseudomonas aeruginosa*: a phenolate siderophore receptor homologous to hydroxamate siderophore receptors. *J. Bacteriol.* **176**, 307-319, doi:10.1128/jb.176.2.307-319.1994 (1994).

23 Bitter, W., Marugg, J. D., de Weger, L. A., Tommassen, J. & Weisbeek, P. J. The ferric-pseudobactin receptor PupA of *Pseudomonas putida* WCS358: homology to TonB-dependent *Escherichia coli* receptors and specificity of the protein. *Mol. Microbiol.* **5**, 647-655, doi:10.1111/j.1365-2958.1991.tb00736.x (1991).

24 Koebnik, R., Hantke, K. & Braun, V. The TonB-dependent ferrichrome receptor FcuA of *Yersinia enterocolitica*: evidence against a strict co-evolution of receptor structure and substrate specificity. *Mol. Microbiol.* **7**, 383-393, doi:10.1111/j.1365-2958.1993.tb01130.x (1993).

25 Kenney, C. D. & Cornelissen, C. N. Demonstration and characterization of a specific interaction between gonococcal transferrin binding protein A and TonB. *J. Bacteriol.* **184**, 6138-6145, doi:10.1128/jb.184.22.6138-6145.2002 (2002).

26 Burkhard, K. A. & Wilks, A. Characterization of the outer membrane receptor ShuA from the heme uptake system of *Shigella dysenteriae*. Substrate specificity and identification of the heme protein ligands. *J. Biol. Chem.* **282**, 15126-15136, doi:10.1074/jbc.M611121200 (2007).

27 Balhesteros, H. *et al.* TonB-dependent heme/hemoglobin utilization by *Caulobacter crescentus* HutA. *J. Bacteriol.* **199**, e00723-16, doi:10.1128/JB.00723-16 (2017).

28 Benevides-Matos, N., Wandersman, C. & Biville, F. HasB, the *Serratia marcescens* TonB paralog, is specific to HasR. *J. Bacteriol.* **190**, 21-27, doi:10.1128/JB.01389-07 (2008).

29 Katayama Y, N. S., Nakamura M, Yano K, Yamasaki M, Morohoshi N, Haraguchi T. Cloning and

expression of *Pseudomonas paucimobilis* SYK-6 genes involved in the degradation of vanillate and protocatechuate in *P. putida*. *Mokuzai Gakkaishi* **33**, 77-79 (1987).

30 Fujita, M. *et al.* A TonB-dependent receptor constitutes the outer membrane transport system for a lignin-derived aromatic compound. *Commun. Biol.* **2**, 432, doi:10.1038/s42003-019-0676-z (2019).

31 Sonoki, T. *et al.* Glucose-free *cis*,*cis*-muconic acid production via new metabolic designs corresponding to the heterogeneity of lignin. *ACS Sustainable Chem. Eng.* **6**, 1256-1264, doi:10.1021/acssuschemeng.7b03597 (2018).

32 Bolivar, F. & Backman, K. Plasmids of *Escherichia coli* as cloning vectors. *Methods Enzymol.* **68**, 245-267, doi:10.1016/0076-6879(79)68018-7 (1979).

33 Studier, F. W. & Moffatt, B. A. Use of bacteriophage T7 RNA polymerase to direct selective high-level expression of cloned genes. *J. Mol. Biol.* **189**, 113-130, doi:10.1016/0022-2836(86)90385-2 (1986).

34 Figurski, D. H. & Helinski, D. R. Replication of an origin-containing derivative of plasmid RK2 dependent on a plasmid function provided in *trans*. *Proc. Natl. Acad. Sci. USA* **76**, 1648-1652, doi:10.1073/pnas.76.4.1648 (1979).

35 Blatny, J. M., Brautaset, T., Winther-Larsen, H. C., Karunakaran, P. & Valla, S. Improved broad-host-range RK2 vectors useful for high and low regulated gene expression levels in Gram-negative bacteria. *Plasmid* **38**, 35-51, doi:10.1006/plas.1997.1294 (1997).

36 Kaczmarczyk, A., Vorholt, J. A. & Francez-Charlot, A. Markerless gene deletion system for Sphingomonads. *Appl. Environ. Microbiol.* **78**, 3774-3777, doi:10.1128/AEM.07347-11 (2012).

37 Silva-Rocha, R. *et al.* The Standard European Vector Architecture (SEVA): a coherent platform for the analysis and deployment of complex prokaryotic phenotypes. *Nucleic Acids Res.* **41**, D666-675, doi:10.1093/nar/gks1119 (2013).

38 Sievers, F. *et al.* Fast, scalable generation of high-quality protein multiple sequence alignments using Clustal Omega. *Mol. Syst. Biol.* **7**, 539, doi:10.1038/msb.2011.75 (2011).
